# Surgically Induced Digital Distal Syndactyly for Prevention of Digital Growth Deformities Around the Joints: A New Technique

**DOI:** 10.5812/traumamon.7338

**Published:** 2012-10-10

**Authors:** Shahram Nazerani, Mohammad Hosein Kalantar Motamedi, Aydin Pirzeh, Jalal Vahedian, Tara Nazerani, Tina Nazerani

**Affiliations:** 1 Department of Surgery, Tehran University of Medical Sciences, Firuzgar Medical Center, Tehran, IR Iran; 2 Trauma Research Center, Baqiyatallah University of Medical Sciences, Tehran, IR Iran

**Keywords:** Syndactyly, Therapeutics, Contrature of Joints Surgery

## Abstract

**Background:**

Correction of digit deformities at or near the Joints is performed easily ; however, maintaining the result is often difficult either due to noncompliance of the patient to wear the postoperative splints or problems related to unequal growth of bones or normal tissues compared to the scarred or operated side.

**Objectives:**

The aim of this study was to overcome the above mentioned problems for which we propose the "Distal d Digit Syndactyly" technique.

**Materials and Method:**

This method is based on the concept of suturing the distal phalanx of the deformed digit to the normal adjoining finger to help prevent the recurrence of the anomaly during the child’s growth period or the very important three or four postoperative months of scar maturation in the adult. After the correction of deformity of the finger or toe, "Distal Syndactyly" is created by two flaps on the adjoining digits; one base is dorsally hinged and the other one volar and after elevating the flaps they are sutured together. During the three postoperative weeks care is taken that this attachment is not disrupted and after healing a "distal syndactyly" is created which is very durable and in children it stretches with growth and does not impede the digit’s growth.

**Results:**

Eleven patients with congenital and traumatic digit anomalies were treated. The recurrence of the problem was prevented in 9 patients; in 2 patients with intact Syndactyly the contracture recurred by stretching the Syndactyly skin. The period of the “Joining” ranged from 6 months to three years and cosmetic appearance was acceptable to the patient and parents.

**Conclusion:**

This technique by joining a deformed digit to a normally growing adjacent digit prevents the postoperative recurrence of the contracture or growth induced deviation in the digits of noncompliant patients especially children.

## 1. Background

In some cases of congenital or traumatic diseases of the fingers and toes, the corrected deformity recurs in a few weeks or months due to ongoing healing or growth processes, i.e. child's growth or the healing process or scar formation leads to deviation of the digit at the joints. Even with diligent postoperative visits, physiotherapy and splints, there is a small group of patients who are noncompliant or unequal or unbalanced growth of the bone or scar lead to recurrence of the contracture or deviation of the joint. The disease process usually involves soft tissues and ligaments around the joints and in a growing child which in time leads to deviation of not only joints but also the bones. The fear of damage to the growth plate prevents an osteotomy near the plate to completely correct a deviated finger or toe. Although orthoses and night splints help prevent the disease process, in longstanding conditions such as flexion contractures and congenital disease processes such as overriding or the duplication of toes, a soft tissue deviation is seen which in time will in children lead to bony deformity when not corrected. The orthoses which prevent the recurrence of deviation or contracture are cumbersome and very difficult to keep on for long periods of time especially in small children (1-5).

Patients with burn or trauma contractures in one finger especially the little finger with deviated and deformed joints decline amputation and insist on saving the finger at all costs. After the soft tissue distraction which corrects the deformity and completion of coverage, there must be a long-term use of orthoses to hold the joint and soft tissues in this “corrected” position. The orthoses are usually not able to hold this position are cumbersome and in small children are usually removed by a restless child during sleep, thus this "temporary measure" is not enough and some form of long-term and easier method must be used to prevent recurrence (2, 6).To overcome this problem we present a novel approach by creating a "distal digit Syndactyly" (DDS). In this method we surgically create a distal Syndactyly between two adjoining fingers or toes and when this "joining" heals the joined fingers function together and the normal finger acts as a "Biologic Buddy Taping" to hold the operated finger in normal position and also helps in the return to normal function of the diseased digit. Eleven cases of upper and lower extremity digit problems are presented and the advantages of this technique are discussed in detail.

## 2. Objectives

The aim of this study was to overcome the recurrence of joint deformity of the fingers and toes after corrective surgery by the application of "Distal Digit Syndactyly".

## 3. Materials and Methods

The patients were 5 females and 6 males. Congenital disorders comprised 8 cases or 87% of the group. Complex syndactyly was seen in the majority of congenital cases with several symbrachydactyly patients needing multiple procedures with joint problems. The DDS technique is a safe and reversible method with minimal scarring and can be maintained indefinitely. In designing the flaps it is important to take into consideration that since the two fingers are not of the same length, the flaps should be designed in complete extension of the fingers so the shorter finger cannot bend the longer one. The indications of this technique are to act as a biologic “Buddy Taping” procedure; and on the other hand by attaching a digit which has had an operation on the joint or near the growth plate, to a normally growing straight digit to help the diseased digit grow straight. The anomalies in lower extremities are: overriding toes, recurrence of deviation in operated congenital bifid toes; in the hand: complex syndactyly with PIP Joint collateral ligament defects, in which with time, the scarring causes deviation of the finger at the PIP joint which is another indication for this technique.

### 3.1. Technique

**Figure 1:**
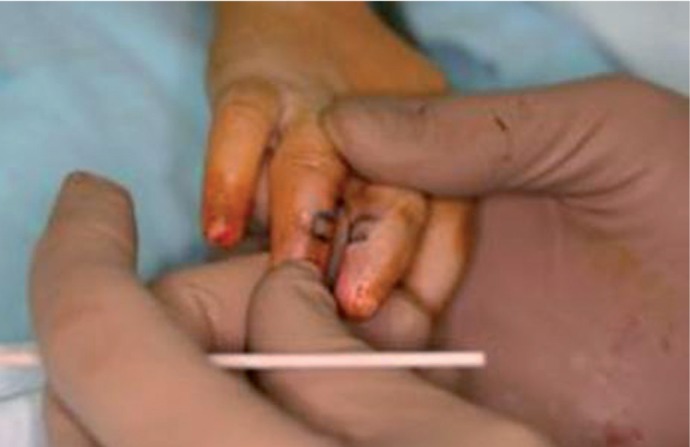
A Typical Case Requiring Treatment

**Figure 2:**
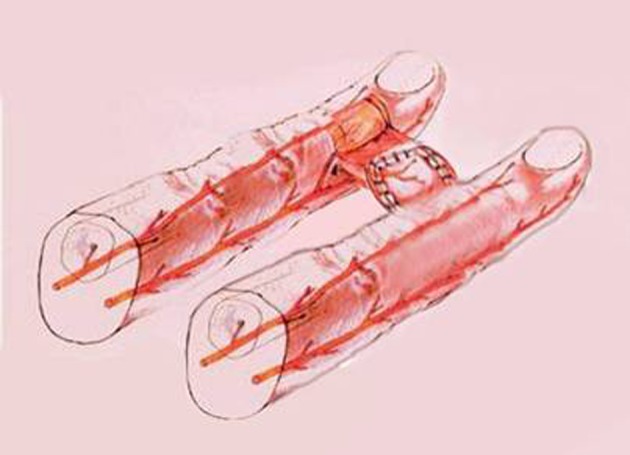
A Schematic Diagram of the Procedure

The technique is simple; two rectangular flaps are designed and marked on the adjoining fingers, one being the operated one and the other the normal digit. The flaps are designed in a way that the bases are in opposite planes, i.e., one flap’s base is volar and the other flap’s base is dorsal (Figures 1, 2). It is important to hold the fingers straight during the design and marking so that the flaps reach each other at exactly the same level; this prevents the future bending of the longer finger.

**Figure 3:**
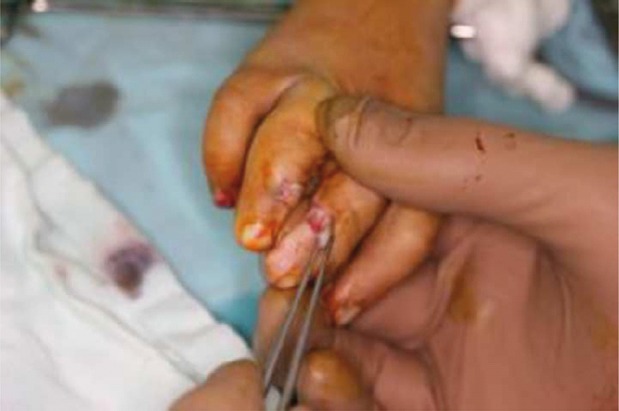
Flaps mobilized.

**Figure 4:**
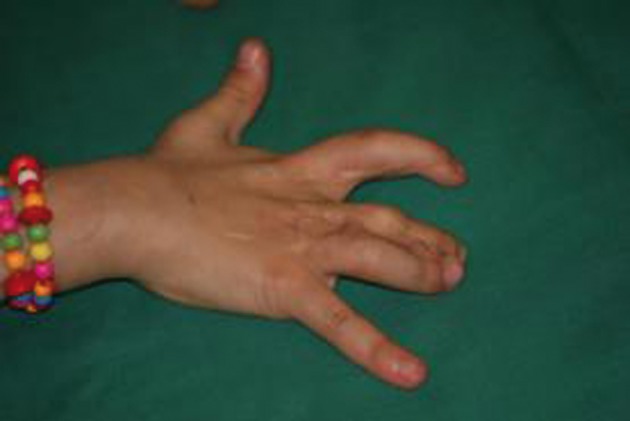
Postoperative result

**Figure 5:**
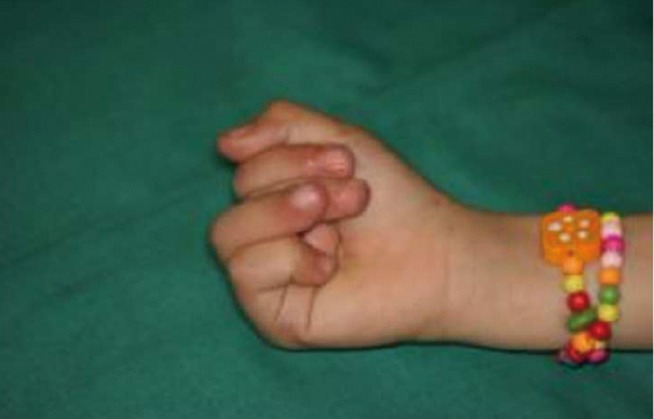
Flexion

**Figure 6:**
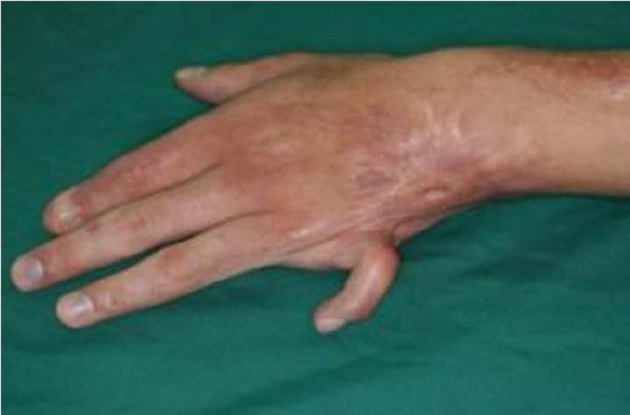
Another patient with contracture of the small finger.

The flaps are elevated carefully under magnification to prevent damage to the vascular and neural structures beneath them. The flaps are then joined by absorbable sutures (Figures 3, 4). The donor raw surfaces on the fingers are left to heal and no closure or skin graft is needed. After the wounds heal, the Syndactyly acts as a living orthosis or "Buddy taping" preventing the recurrence of contractureor joint deformity and helps the operated digit's function by the movements of the normal digit(Figures 5, 6). During the first few postoperative weeks care is taken that this „jointing‟ is not disrupted and after healing a "distal Syndactyly" is created which is very durable. It is noteworthy that with time the Syndactyly stretches and does not bend the longer finger. During the first two weeks the hygiene of the fingers is a bit difficult to maintain because of fear of disruption of the joining flaps and must be done by the medical personnel; however, after this time hygiene can be easily maintained by cleaning the web via swabs and the parents can themselves do it. We have not observed any maceration of the web space in this group of patients. We use no Wires whatsoever to hold the flaps together and usually it takes around two weeks for the flap to heal,during this time the digits should be taped to prevent flap separation.

**Figure 7:**
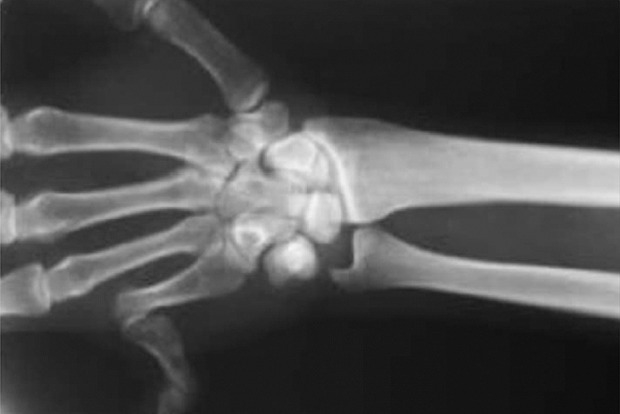
Radiograph of the small finger

From 2000 to 2010 in a period of ten years, 11 patients with different diseases of digits in the upper and lower extremities were treated by this technique(Table 1).DDS was applied to the cases of long standing contracture or recurrent surgically corrected deformities(Figure 7).

**Table 1:** Demographic Data of the Patients

Age, Y	Sex	Injury	Extremity Involved	Complication
**2**	F	Congenital Complex Syndactyly	Upper Extremity	-
**8**	M	Trauma Meat Grinder injury	Upper Extremity	-
**45**	M	Trauma Gun Shot wound	Upper Extremity	-
**3**	F	Congenital Complex Syndactyly	Upper Extremity	-
**8**	M	Congenital Short Little finger Metacarpal	Upper Extremity	Partial necrosis of created flaps
**25**	M	Trauma Burn contracture	Upper Extremity	-
**1**	M	Congenital Symbrachydactyly	Upper Extremity	Function is poor
**1.5**	M	Congenital Symbrachydactyly	Upper Extremity	Function is poor
**8 months**	F	Congenital Duplication of Little toe	Lower Extremity	-
**22**	F	Congenital Overriding little Toe	Lower Extremity	-
**8**	F	Congenital Symbrachydactyly	Lower Extremity	-

**Figure 8:**
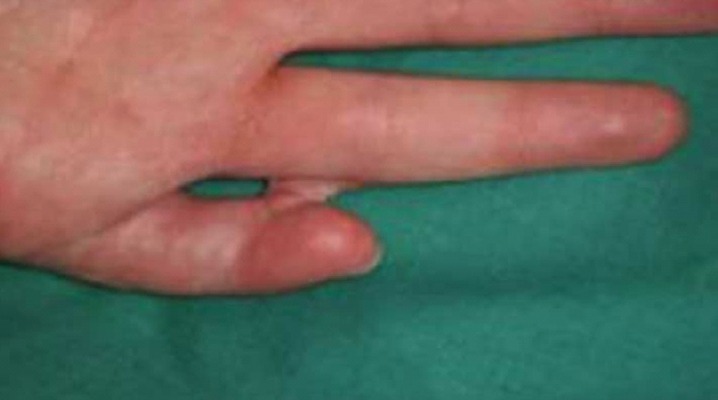
Postoperative result

## 4. Results

**Figure 9:**
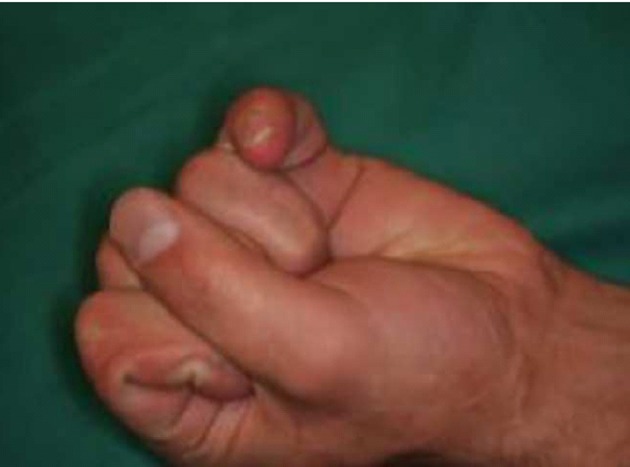
Flexion

After distraction and normalizing the alignment of the finger without any damage to the joint, a skin graft was inserted when needed and to prevent recurrence of MP joint contracture a DDS was created(Figures. 8, 9).

**Figure 10:**
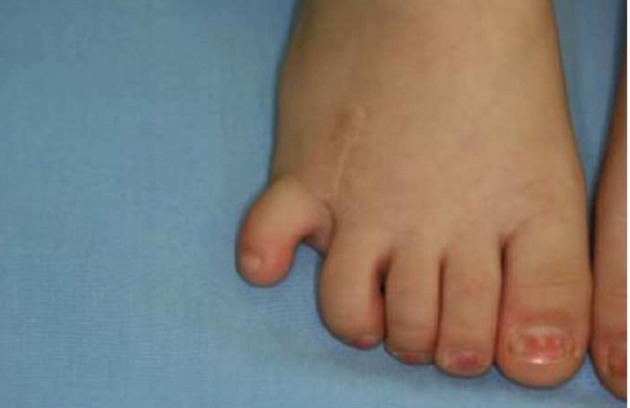
Another patient

**Figure 11:**
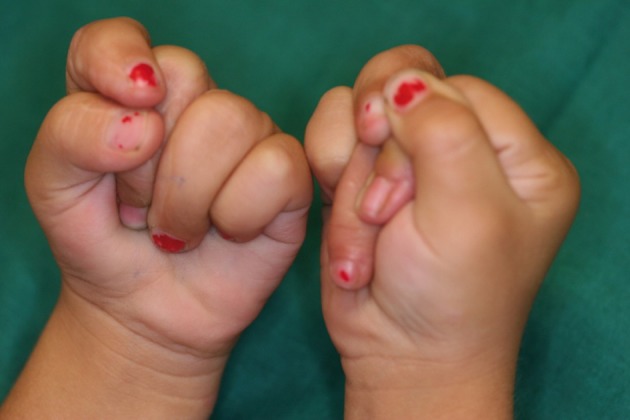
A child with complex syndactyly after the operation and overriding fingers in flexion

**Figure 12:**
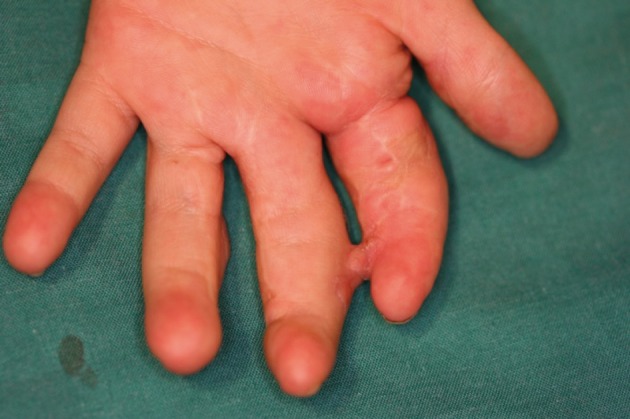
Distal syndactyly (volar aspect)

**Figure 13:**
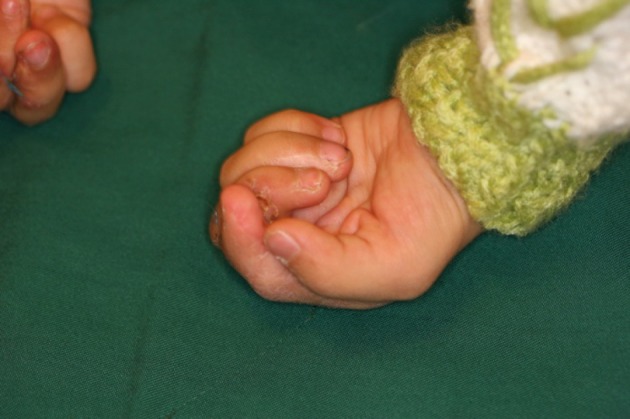
The fingers are flexed and the overriding has been corrected

This procedure was used in a bilateral little toe duplication. The medial side duplicated toe was removed and reconstruction was performed. After 8 months, the parents were concerned about the deviation of the operated toe which in their opinion was progressing (Figure 10). DDS was performed by joining the little toe distally to the fourth normal toe. The result at three months was very good and the parents were satisfied. Results of another case are shown in Figures 11-12-13. The correction of deformity which may involve the ligaments of the MP joints due to longstanding tight footwear can also be corrected via DDS.

## 5. Discussion

In congenital or traumatic diseases of the digits, there is a subset of patients who are either noncompliant to wear the orthosis or the disease process itself leads to recurrence of the digit contracture or joint deviation. The proposed methods such as long term splints are not able to prevent an ongoing disease process and sometimes a child with congenital disorder of the hand such as syndactyly or symbrachydactyly needs long sessions of physiotherapy and splinting which, in a frustrated parent and noncompliant child may be tiresome and leads to a drop out. A desperate mother once complained: "Doctor I am tired of coming and going and taping the fingers together, can you do something to suture the fingers together? When these problems were encountered at our hand clinic we came up with the idea of "Biologic Buddy Taping" or "Distal Digit Syndactyly." This is created via soft tissue joining of the distal part of the fingers or toes which prevents recurrence of deformities as the normal digit helps the operated one regain function and remain straight. In a paper by Hahn et al the authors use a cross finger flap to treat the volar contracture defect. They observed that this flap prevented recurrence and increased ROM (6).

The distal syndactyly is hidden from view in flexion especially in the lower extremity when socks are worn. Additionally, shoes are easily worn whereas in the lower extremity it is not possible to wear shoes and splint together. In this case series the parents and patients were satisfied with the results. The "distal digit syndactyly" seems to be a reliable technique to prevent the postoperative recurrence of the disease in the digits of noncompliant patients or in a growing child. The syndactyly created is easily accepted by the patient and can endure the wear and tear of daily life and the patient can easily participate in sports activities. The take down of the syndactyly is quite simple and can be done on an outpatient basis. The take-down of the syndactyly was done from 6 months to three years.
